# Fabrication and Plasmonic Characterization of Metasurfaces Patterned via Tunable Pyramidal Interference Lithography

**DOI:** 10.3390/mi17010104

**Published:** 2026-01-13

**Authors:** Saim Bokhari, Yazan Bdour, Ribal Georges Sabat

**Affiliations:** Department of Physics and Space Science, Royal Military College of Canada, Kingston, ON K7K 7B4, Canada

**Keywords:** pyramidal interference lithography, metasurfaces, surface plasmon resonance, surface plasmon resonance imaging, tunable periodic structures, azobenzene thin films

## Abstract

Large-area metasurfaces were fabricated via a tunable pyramidal interference lithography (PIL) technique, which uses custom-built 2-faced, 3-faced, and 4-faced pyramidal prisms to create metasurfaces with customizable nano- and micro-scale surface feature periodicities. The 2-faced prism produced linear surface relief diffraction gratings, while the 3-faced prism produced metasurfaces with triangular lattices and the 4-faced prism produced metasurfaces with square lattices, all on azobenzene thin films. A double inline prism set-up enabled control over the metasurface feature periodicity, allowing systematic increase in the pattern size. Additional tunability was achieved by placing a prism inline with a lens, allowing precise control over the metasurface feature periodicity. A theoretical model was derived and successfully matched to the experimental results. The resulting metasurfaces were coated with gold and exhibited distinct surface plasmon resonance (SPR) and surface plasmon resonance imaging (SPRi) responses, confirming their functionality. Overall, this work establishes PIL as a cost-effective and highly adaptable metasurface fabrication method for producing customizable periodic metasurfaces for photonic, plasmonic, and sensing applications.

## 1. Introduction

Metasurfaces are ultrathin engineered two-dimensional materials composed of repeating micro- or nano-scale surface features that have revolutionized the way electromagnetic waves can be manipulated at subwavelength scales [1,2]. They allow for precise control over the wavelength [3], amplitude [4], phase [5], and polarization [6] of electromagnetic waves, including light, which has led to applications such as meta-lenses [7], holography [8], imaging [9], and advanced sensing technologies [10]. The ability of metallic metasurfaces to guide and manipulate electromagnetic fields at their boundary with other dielectric materials also makes them highly relevant to surface plasmon resonance (SPR) sensing and surface plasmon resonance imaging (SPRi) [11]. These phenomena rely on the metallic metasurface to couple incident electromagnetic waves into surface plasmon modes at the metal/dielectric interface [12]. When compared to simple gratings, metasurfaces can significantly enhance, the SPR coupling efficiency [13], field confinement [14], and polarization tunability [15]. They are especially attractive for SPR and SPRi applications due to their ability to induce multidirectional plasmonic modes [16,17], which are useful in imaging applications where spatial contrast is required [18,19]. Despite these advantages, the fabrication of large-area and high-quality metasurfaces remains challenging. Conventional techniques such as electron beam lithography, nanoimprint lithography, and focused ion beam milling offer excellent manufacturing resolution but are expensive, slow, and difficult to scale to practical devices [20,21,22,23].

Interference lithography provides an interesting alternative for the fabrication of photonic metasurfaces because it permits the creation of large-area periodic surface patterns in a single laser beam exposure [24]. Amongst the various methods of interference lithography, pyramidal interference lithography (PIL) is particularly attractive since it uses pyramidal prisms to achieve complex light interference patterns with controllable surface feature periodicity [25]. Azobenzene-based thin films are known to undergo photoisomerization, which leads to molecular reorganization upon laser illumination. Repeated trans–cis–trans isomerization cycles under spatially varying light intensity and polarization drive directed molecular motion, resulting in photoinduced surface relief formation [26]. This mass transport occurs predominantly from regions of high irradiance toward regions of lower irradiance, enabling stable replication of the optical interference pattern as a surface topography on the azobenzene film [27]. When combined with PIL, this enables the creation of large-area and uniform surface features on the azobenzene films [28], mimicking the laser interference pattern generated by the pyramidal prism. Although PIL was previously used to create metasurfaces [25], their surface feature periodicity was bound to the fixed pyramidal prism geometry and could only be changed by using a completely different prism with a different base angle. In addition, the surface plasmon resonance response from these metasurfaces has not been previously studied.

In this work, triangular and square metasurface patterns, with 3-fold and 4-fold symmetries, were inscribed on azobenzene thin films using two inline pyramidal prisms, thus enabling control of the resulting metasurface feature periodicity. Furthermore, a bi-convex lens was placed after a pyramidal prism in the inscription set-up to magnify the laser interference pattern and achieve tunable micro-scale surface patterning. The resulting metasurfaces closely matched Python-ran simulations, thus confirming the reliability of this method. Finally, surface plasmon resonance spectroscopy and imaging was conducted on the resulting gold-coated metasurfaces. Overall, this work establishes PIL as a scalable and cost-effective method for fabricating large-area plasmonic metasurfaces with controllable geometry. We report for the first time on the excitation of surface plasmon resonance and surface plasmon resonance imaging on metasurfaces created using PIL on azobenzene films. This study highlights the strong potential of this technique for future imaging and sensing technologies based on metasurfaces.

## 2. Numerical Analysis

**Figure 1:**
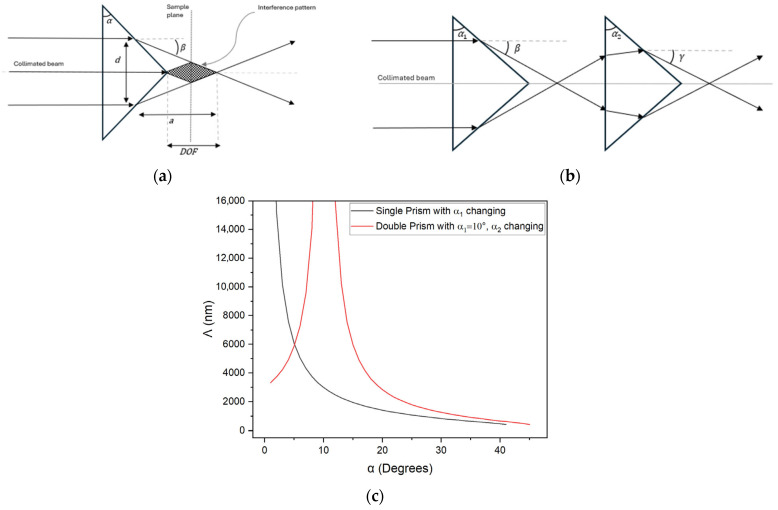
(**a**) Schematic of the single bi-prism PIL configuration. A collimated laser beam of diameter d enters a bi-prism with base angle α, producing two refracted beams at angle β which interfere within the DOF to generate an interference pattern. (**b**) Schematic of the double bi-prism PIL configuration, where the refracted beams from the first bi-prism enter a second bi-prism and are further refracted at angle γ. (**c**) Plot of the calculated grating pitch *Λ* versus the bi-prism base angle. The black curve shows the single bi-prism set-up. The red curve shows the double bi-prism set-up with a fixed $\alpha_{1}=10{}^{\circ}$ and varying $\alpha_{2}$.

When a collimated laser beam is incident on a pyramidal prism, the relationship between the prism geometry and the resulting light interference pattern can be determined through a geometrical analysis of the prism parameters. As represented in Figure 1a, a collimated laser beam with wavelength λ is incident on a 2-faced bi-prism with base angle α. Due to refraction, the light exits the prism at angle β, which is dependent on α and the refractive index of the prism material $n_{p}$. Using Snell’s law, β can be expressed as [25]

(1) $$\beta =\sin^{-1}\left(n_{p}\sin\left(\alpha \right)\right)-\alpha$$

The depth of focus is defined as the area where the two exiting light beams interfere and form a sinusoidal interference pattern with constant grating pitch Λ, as determined by

(2) $$\Lambda =\frac{\lambda }{2\sin\left(\beta \right)}$$

The dependence of Λ on base angle α for a pyramidal bi-prism is plotted in Figure 1c as the black curve. For a given base angle, only a single pitch value can be achieved, which limits the ability of the experimental set-up to produce different metasurface feature periodicities. To expand the range of possible grating pitches without modifying the base angle of the bi-prism, a double bi-prism configuration can be used, as illustrated in Figure 1b. In this configuration, the output beam from the first bi-prism with base angle $\alpha_{1}$ enters the second bi-prism with base angle $\alpha_{2}$, altering the resulting interference pattern. The distance between the two bi-prisms affects the depth of focus interference area but not the interference pattern periodicity. The final refracted angle γ after the second bi-prism can be expressed as

(3) $$\gamma =\left|-\mathrm{si}n^{-1}\left(n_{2}\sin\left(\alpha_{2}-\sin^{-1}\left(\frac{\sin\left(\beta \right)}{n_{1}}\right)\right)\right)+\alpha_{2}\right|$$

where $n_{1}$ and $n_{2}$ represent the index of refraction of the first and second bi-prisms, respectively. The absolute value for γ ensures that the calculated pitch remains physically meaningful. The final grating pitch Λ for the double bi-prism configuration is then expressed as

(4) $$\Lambda =\frac{\lambda }{2\sin\left(\gamma \right)}$$

The resulting pitch variation for the double bi-prism configuration is also plotted in Figure 1c as the red curve, where the first bi-prism base angle is fixed at α=10° and the second bi-prism base angle $\alpha_{2}$ is varied. Compared to the single bi-prism configuration, the double bi-prism arrangement provides access to a broader range of metasurface feature periodicities. As illustrated, achieving large micrometer-sized grating periodicities using a single bi-prism requires very small base angles, which are difficult to manufacture. In contrast, the double bi-prism configuration can produce comparable periodicities using two readily available prisms, thereby significantly enhancing the versatility of the PIL technique for metasurface fabrication.

For plasmonic metasurfaces fabricated from these periodic structures, the interference pitch, Λ, determines the spectral position of surface plasmon resonances. The resonance wavelength, $\lambda_{SPR}$, can be estimated using a grating-coupling condition that relates the grating periodicity to the surface plasmon wavevector at the metal–dielectric interface [29]. At normal incidence, this relation can be expressed as

(5) $$\lambda_{SPR}=n\Lambda \left[\sqrt{\frac{\varepsilon_{m}}{\left(n^{2}+\varepsilon_{m}\right)}}\right]$$

where n is the refractive index of the surrounding dielectric and $\varepsilon_{m}$ is the relative permittivity of the metal. This expression provides an estimate of the expected SPR wavelength for shallow, grating-like structures but might applicable to more complex metasurfaces.

Although the double bi-prism configuration expands the achievable pitch range, it remains fundamentally constrained by the fixed base angles of the prisms. To overcome this limitation, a bi-convex lens can be incorporated into the set-up to dynamically adjust the laser interference pattern. Through a similar ray-tracing analysis, when the lens is placed inline after a bi-prism and before the azobenzene film, the resulting interference pattern, and thus the grating pitch, can be magnified depending on the distance between the lens and the azobenzene sample.

The bi-prism interference theory can be further expanded to include not only 2-faced bi-prisms but pyramidal prisms with *N* faces. Two-dimensional interference patterns, analogous to those that could be generated when a collimated laser beam is incident on *N*-faced pyramidal prisms, were simulated using a simple wave-based numerical model reliant on the coherent superposition of monochromatic spherical wavefronts emitted from multiple equidistant point sources and arranged uniformly along the circumference of a circle with radius R. The angular position $\theta_{i}\;$ of each source is given by

(6) $$\theta_{i}=\frac{2\pi i}{N}$$

where N is the total number of sources and i is the index of each source iterating from 0 to N−1. Python-ran (v. 3.8.20) simulations were carried out for single N=3 or N=4 pyramidal prisms, as well as for double inline N=3 or N=4 pyramidal prisms. The spatial coordinates of the sources are defined as

(7) $$x_{i}=R\cos\left(\theta_{i}\right),\;y_{i}=R\sin\left(\theta_{i}\right)$$

Each point source emits a spherical wave with spatial periodicity Λ given by Equations (2) and (4) for the single and double pyramidal prism configurations, using prism base angles of α=30° (for the single prism) and α=10° followed by $\alpha_{2}=30{}^{\circ}$ (for the double prisms). The wavelength λ was set to 488 nm and the contribution of the ith source at an arbitrary observation point $\left(x,y\right)$ is given by

(8) $$E_{i}\left(x,y\right)=\sin\left(2\pi \frac{c}{\lambda }\cdot r_{i}\right)$$

where $r_{i}$ is the Euclidean distance between the observation point and the source, represented as

(9) $$r_{i}=\sqrt{{\left(x-x_{i}\right)}^{2}+{\left(y-y_{i}\right)}^{2}}$$

The total electric field distribution $Z\left(x,y\right)$ resulting from the interference of all N sources is

(10) $$Z\left(x,y\right)=\sum_{i}^{N-1}sin\left(2\pi f\cdot \sqrt{{\left(x-x_{i}\right)}^{2}+{\left(y-y_{i}\right)}^{2}}\right)$$

This simulation enables the visualization and quantitative analysis of the interference patterns formed by multiple light sources, providing insight into field modulation relevant to the pyramidal metasurface fabrication. The total electric field was sampled on a uniform square grid and converted to a normalized irradiance I∈[0,1]:

(11) $$I=\;\;\frac{Z\left(x,y\right)-\min\left\{Z\right\}}{\max\left\{Z\right\}-\min\left\{Z\right\}}$$

To account for the azobenzene molecular photo-dynamics, in which molecules migrate from high- to low-light irradiance regions, the theoretical pattern in Equation (11) was inverted as follows:

(12) $$I_{inv}=\;\;1-I$$

## 3. Materials and Methods

### 3.1. Fabrication of Metasurfaces

Metasurfaces were inscribed on thin films of azobenzene molecular glass using custom-built pyramidal prisms to achieve 3- and 4-fold surface pattern symmetries. Azobenzene molecules undergo photoisomerization upon light exposure, which induces molecular mass transport and film surface reorganization, thus enabling the recording of the light interference pattern onto the azobenzene thin film [30]. Disperse Red 1 azobenzene molecular glass (gDR1) was synthesized following procedures described in the prior literature [31]. A 3 wt.% solution of gDR1 in dichloromethane (DCM) was prepared, and 75 µL was deposited onto a clean Corning 0215 microscope slide (Ted Pella, Redding, CA, USA). The solution was spin-coated at 1400 rpm for 35 s, yielding a uniform film thickness of ~300 nm, measured using a DektakXT stylus profilometer (Bruker, San Jose, CA, USA). Coated slides were baked at 70 °C for 15 min to remove residual solvent.

**Figure 2:**
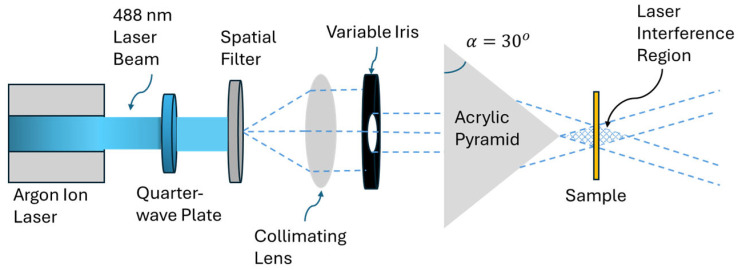
Schematic of the laser exposure set-up using a single pyramid configuration. A 488 nm beam generated by an argon ion laser is made circularly polarized using a quarter-wave plate. A spatial filter and collimating lens shape the beam, followed by a variable iris to adjust beam size. The beam passes through a single pyramidal prism with either *N* = 3 or *N* = 4 faces, generating the interference pattern on the azobenzene-coated sample.

A 95L-MidUV 488 nm continuous-wave argon ion laser (Lexel Laser, Cambridge Laser Laboratories, Fremont, CA, USA) was used for the optical inscription of the metasurfaces. A schematic of the experimental set-up for the single pyramidal prism configuration is shown in Figure 2. The laser beam was first made circularly polarized using a quarter-wave plate, then it passed through a spatial filter and a collimating lens to ensure a uniform beam profile. A variable iris was used to control the beam diameter before it was directed onto custom-built acrylic pyramidal prisms with either *N* = 3 or *N* = 4 faces and a base angle of α=30°. The prisms refracted the incoming beam to generate symmetric interference patterns, which were projected onto the azobenzene-coated substrate placed at the light interference zone. For *N* = 3, the triangular interference zone or the metasurface area was measured to be approximately 10 mm^2^, meanwhile for *N* = 4, the metasurface area was approximately 25 mm^2^. The prisms used in this work were machined in-house from clear acrylic rods using a precision milling machine equipped with a dividing head, followed by rotary lapping and final polishing with 3 µm diamond paste, as previously reported [25].

**Figure 3:**
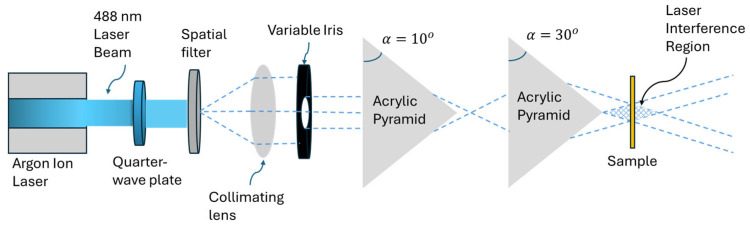
Schematic of the double pyramidal prism set-up. The configuration mirrors the single pyramidal prism set-up but with the addition of a second inline prism. The resulting interference pattern enabled the fabrication of metasurfaces with larger periodicities.

As illustrated in Figure 3, a double pyramidal prism configuration was constructed to expand the range of metasurface surface periodicities, as detailed in the numerical analysis section. The beam emerging from the first prism entered the second prism aligned along the laser beam path. In this configuration and for each exposure, the chosen prism combination had the same number of faces (either *N* = 3 or *N* = 4) but different base angles. For *N* = 3, the triangular interference zone or the metasurface area was measured to be approximately 3 mm^2^, meanwhile for *N* = 4, the metasurface area was approximately 4 mm^2^. This enabled the generation of light interference patterns with periodicities larger than those obtained by using a single multi-faced prism with the same base angle.

Following the laser inscriptions, the resulting metasurfaces were imaged using an atomic force microscope (Dimension Edge AFM, Bruker, San Jose, CA, USA), and the surface profile and modulation depth were obtained using the Nanoscope Analysis V2.0 software. Finally, the metasurfaces were coated with a 50 nm layer of gold using a sputter coater (Q 150V ES Plus, Quorum, Laughton, East Sussex, UK) to enable surface plasmon excitation.

**Figure 4:**
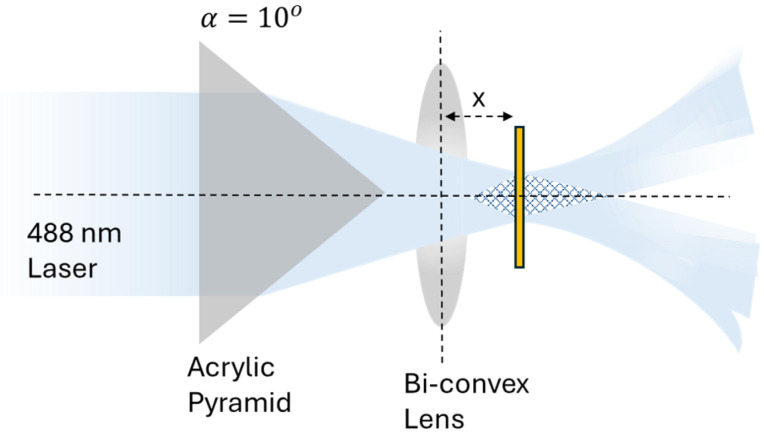
Illustration of the bi-prism–lens configuration. The refracted beam emerging from the bi-prism enters a bi-convex lens placed at distance x.

As illustrated in Figure 4, a third configuration was constructed in which the incoming laser beam was incident on a custom-built pyramidal 2-faced bi-prism, with a base angle α=10°, followed by a bi-convex spherical lens to achieve a continuous tunability of the surface feature periodicity. The lens was positioned at variable distance x from the azobenzene sample, thus magnifying the interference pattern before it was incident onto the azobenzene film. By adjusting the sample-to-lens distance x, the resulting grating pitch could be tuned dynamically during fabrication. Using a HeNe laser and a precise rotary stage, the first-order diffraction beams from each resulting grating from this fabrication set-up were measured to find the exact value of the pitch for each grating.

### 3.2. Surface Plasmon Measurements

**Figure 5:**
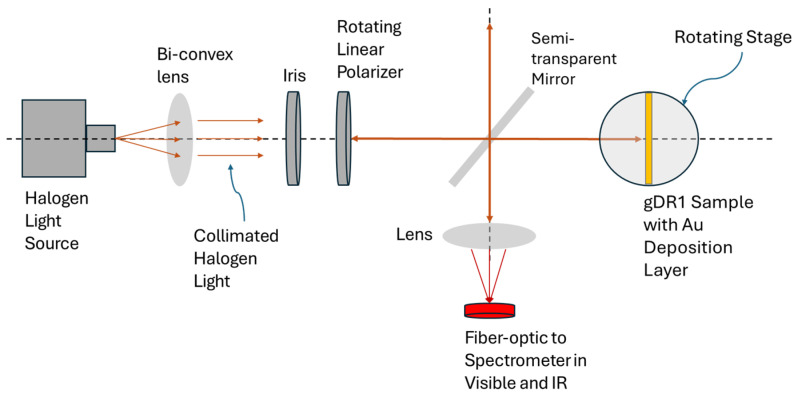
Experimental set-up for the SPR spectroscopy in reflection mode. A stabilized white-light source is collimated, polarized, and directed onto the metasurface through a semi-transparent mirror. The reflected beam is collected and analyzed via spectrometer to resolve SPR behavior.

To characterize the plasmonic response of the metasurfaces produced with the 3-faced and 4-faced pyramidal prisms, the reflection spectra were obtained using the experimental set-up illustrated in Figure 5. The metasurfaces were illuminated with a stabilized halogen white-light source (SLS301, Thorlabs, Newton, CA, USA). The emitted light was collimated using a bi-convex lens and passed through a variable iris to control the beam diameter. The light was then made linearly polarized and incident on a semi-transparent mirror which enabled simultaneous illumination of the sample and redirection of the normally reflected light towards a fiber-optic cable connected to a spectrometer (QE Pro and NIR, Ocean Insight, Orlando, FL, USA). Spectra were normalized by referencing to a flat gold-coated substrate measured under identical illumination and polarization conditions. This configuration enabled measurements of the SPR response in reflection mode under various linear light polarizations.

**Figure 6:**
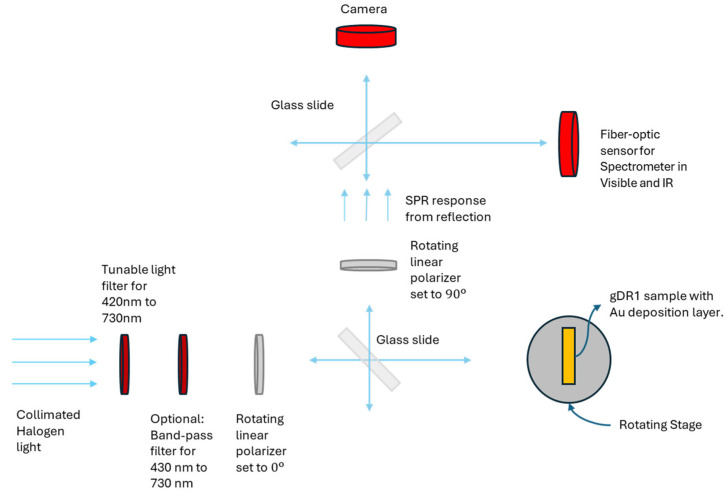
Experimental set-up for SPR imaging in reflection. The configuration incorporates orthogonal polarizers before and after reflection.

As shown in Figure 6, SPR imaging was obtained when the metasurface was placed in between crossed polarizers in order to isolate and image the SPR phenomena to enhance the contrast [32]. The white-light source was first collimated and filtered to select the desired imaging wavelength band using a tunable bandpass filter (K2XL1 Kurios Tunable Filter, Thorlabs, Newton, CA, USA). The filter provides wavelength selection across the visible range from 430 to 730 nm; however, wavelengths beyond 730 nm are not actively filtered and therefore are transmitted onto the sample. The beam then passed through a vertically aligned linear polarizer before illuminating the metasurface. The reflected light from the metasurface was re-directed by a semi-transparent mirror and subsequently passed through a second linear polarizer, oriented orthogonally to the first. Only light that was re-emitted due to the SPR excitation would change its polarization status and be transmitted through the second polarizer and collected by a near-infrared CMOS camera (G-130 TEC1 Goldeye, Allied Vision, Stadtroda, Thuringia, Germany).

## 4. Results

**Figure 7:**
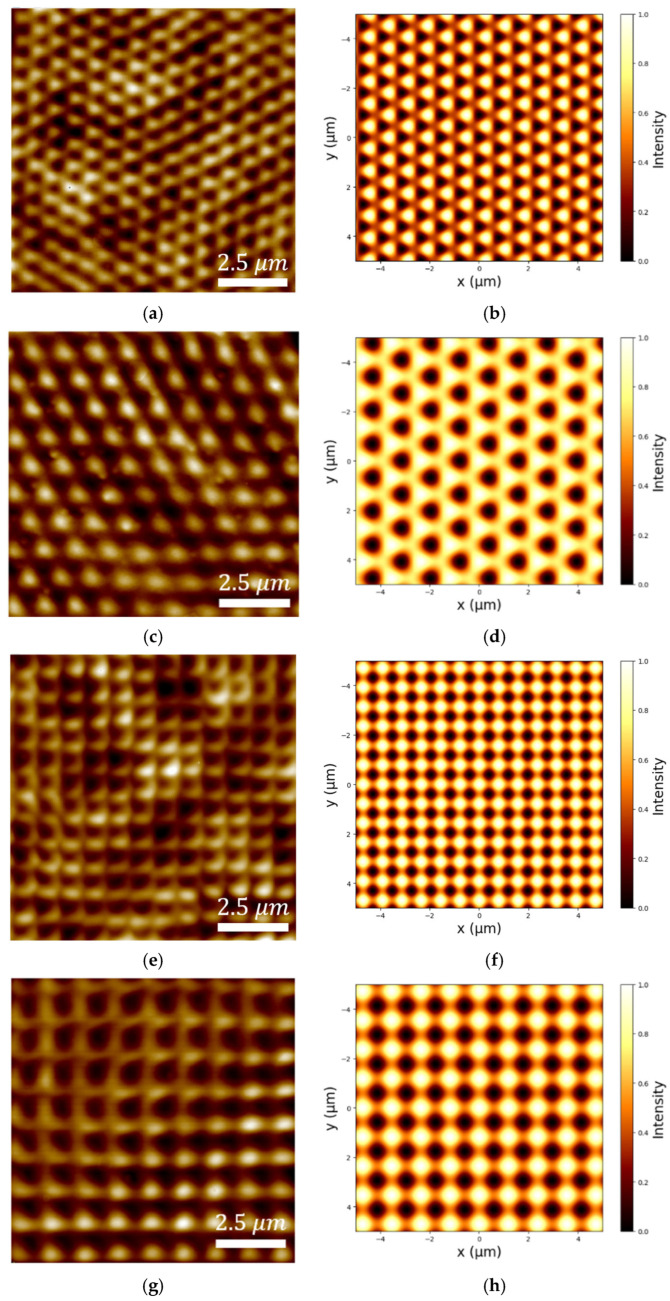
AFM images and their corresponding simulations of metasurfaces patterned using (**a**,**b**) single 3-faced, (**c**,**d**) double 3-faced, (**e**,**f**) single 4-faced, and (**g**,**h**) double 4-faced pyramidal prisms.

The topographical AFM scans of the produced metasurfaces and their corresponding Python-simulated interference patterns are presented in Figure 7 for the single and double 3-faced and 4-faced prism configurations. The theoretical simulations show the spatial light intensity distributions generated by each prism geometry according to Equation (11). The AFM images of the fabricated metasurfaces demonstrate very strong agreement between the theoretically-predicted and experimental surface features. The single pyramidal prism set-up produced triangular patterns with a periodicity of 682 nm when using the 3-faced prism and square patterns with a periodicity of 831 nm when using the 4-faced prism, as shown in Figure 7a,e. The double pyramidal prism configuration produced triangular patterns with a periodicity of 968 nm and square patterns with a periodicity of 1104 nm when using the 3-faced or 4-faced prisms, respectively, as shown in Figure 7c,g.

**Figure 8:**
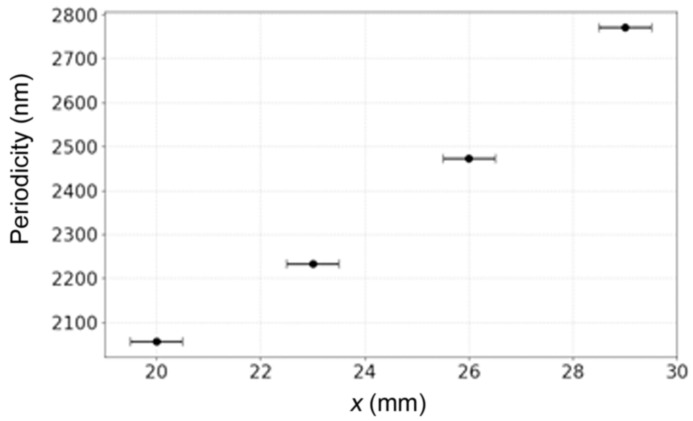
A graph showing the lens distance away from the sample surface, x, which would result in increased grating periodicity using the bi-prism–lens fabrication configuration.

The bi-prism–lens configuration was used to enable the fabrication of different pitch gratings only by varying the distance x between the lens and the azobenzene film, as shown in Figure 8. With the lens positioned 20 mm from the azobenzene surface, the resulting grating pitch was 2050 nm. Incrementally translating the lens away from the sample in 3 mm steps increased the grating periodicity, reaching 2750 nm at a distance of 29 mm. These periodicities exceed the detection range of the SPR spectrometer; thus, these samples were not gold-coated, and SPR measurements were not performed.

**Figure 9:**
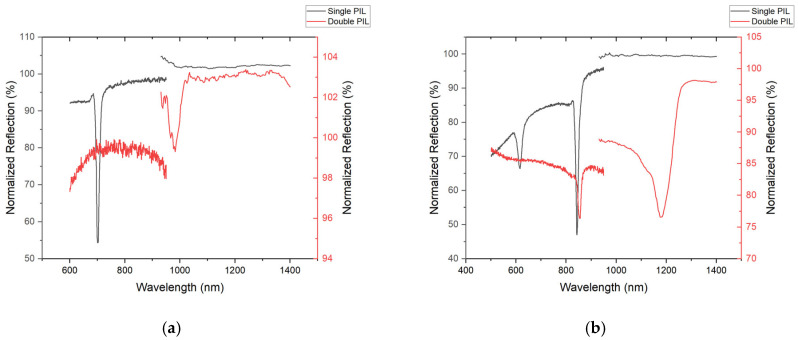
SPR measurements taken by the spectrometer for the metasurface produced with the (**a**) 3-faced pyramidal prism and (**b**) 4-faced pyramidal prism. Spectral readings from metasurfaces patterned from a single prism are shown in black and those patterned with double prisms are shown in red.

The resulting metasurfaces, fabricated using the 3-faced and 4-faced single and double prism configurations, were subsequently coated with gold to achieve surface plasmon resonance. Figure 9 shows the spectroscopic reflection spectra from these metasurfaces utilizing the experimental set-up shown in Figure 5. They all exhibit plasmonic modes, characterized by negative peaks in the reflection spectrum, indicating enhanced transmission through the samples or increased transparency of the gold-coated metasurfaces due to the plasmon resonance. It is noteworthy that the single 4-faced pyramidal prism set-up supported two resonances across the visible spectrum, as seen in the black curve in Figure 9b and as will be explained later.

The double pyramidal prism configuration, with either *N* = 3 or *N* = 4, was used to magnify the interference pattern and to increase the pattern periodicity, as previously shown. The spectral measurements from the metasurfaces patterned using the double prism set-up exhibited red-shifted plasmonic responses associated with the increased pattern periodicity [33].

**Figure 10:**
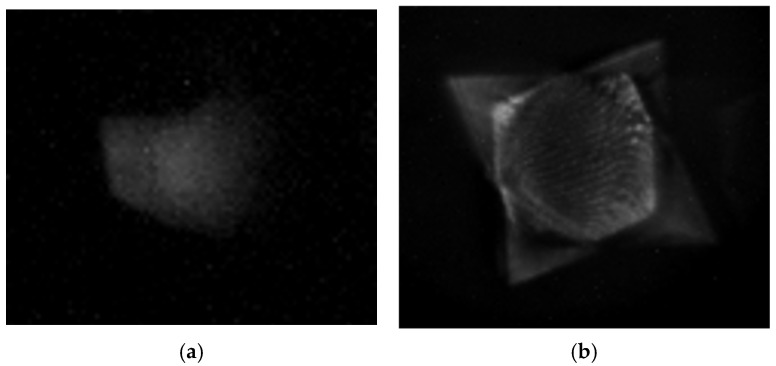
SPR images captured under crossed polarization conditions from metasurfaces fabricated with (**a**) 3-faced and (**b**) 4-faced pyramidal prisms.

The recorded SPRi images, presented in Figure 10, were obtained from the experimental set-up described in Figure 6 and demonstrate localized plasmonic activity on the metasurfaces fabricated with the single 3-faced or 4-faced prism configurations. The triangular metasurface obtained from the single 3-faced prism was imaged using illumination from 700 nm wavelength-filtered light, corresponding to its plasmonic wavelength as shown in Figure 10a. In contrast, the square metasurface fabricated from the single 4-faced prism was imaged using illumination from 615 nm wavelength-filtered light, as shown in Figure 10b. It is important to note that the tunable bandpass filter used in the SPRi set-up operates within the 430–730 nm range and does not block wavelengths beyond 730 nm. As a result, higher-wavelength plasmonic contributions are also present in the acquired image of the metasurface fabricated with the 4-faced prism, consistent with the additional resonance peak observed in the corresponding reflection spectrum from Figure 9b. Despite this limitation, these results provide a visual map of localized plasmonic resonance by the metasurfaces and showcase that the surface feature symmetry affects their plasmonic response.

## 5. Discussion

The experimental results confirm that the single, double, and bi-prism–lens pyramidal fabrication set-ups successfully produced well-defined periodic gratings (for *N* = 2 bi-prism) and metasurfaces (for *N* = 3 and *N* = 4 prisms) that were reproducibly obtained across multiple independently fabricated samples using identical exposure conditions. AFM measurements confirm that metasurfaces produced using the double pyramidal prisms set-up exhibited larger periodicities when compared with those fabricated using a single pyramidal prism set-up. For the 3-faced prism, the triangular lattice periodicity increased from 682 nm (when using a single prism) to 968 nm (when using a double prism), while for the 4-faced prism, the square lattice periodicity increased from 831 nm (when using a single prism) to 1104 nm (when using a double prism). Using these experimentally measured periodicities, the surface plasmon resonance (SPR) wavelengths were estimated via the grating-coupling relation given in Equation (5). For the triangular lattice generated using the three-faced prism, the estimated SPR wavelength increases from approximately 705 nm in the single-prism configuration to 981 nm in the double-prism configuration. Similarly, for the square lattice produced by the four-faced prism, the estimated SPR wavelength shifts from 847 nm to 1115 nm when transitioning from a single- to a double-prism configuration. These observations demonstrate that the double-prism arrangement can systematically modify the interference pattern and thereby control the resulting metasurface feature periodicity.

The single and double pyramidal prism configurations are sufficient for producing uniform and highly periodic metasurfaces. However, fabricating custom pyramidal prisms to obtain different periodicities is not always practical or cost-effective. The bi-prism–lens set-up addresses this limitation by offering a continuous and dynamically tunable pitch range. In this set-up, the bi-prism determines the symmetry and angular distribution of the interfering beams, while the lens controls the spatial scaling of the interference pattern through optical magnification. As observed in Figure 8, by increasing the distance between the lens and the azobenzene sample along the beam path, the magnification factor from the lens changes continuously, enabling an incremental increase in the grating pitch. Experimentally, a 2-faced bi-prism (*N* = 2) was used to verify this concept. When the lens-to-sample distance was 20 mm, the grating pitch was 2050 nm. Increasing the lens-to-sample distance in 3 mm increments progressively increased the grating pitch, reaching a value of 2750 nm at 29 mm. This provides a straightforward method for tuning the grating pitch without requiring additional prisms, significantly enhancing the flexibility of the PIL technique. Furthermore, the resulting metasurfaces from the 3-faced and 4-faced pyramidal prisms could equally be modified by placing a lens after those prisms, thus magnifying the resulting interference pattern.

The fabricated metasurfaces were tested for supporting surface plasmon resonance modes as shown in Figure 9. For the single-prism metasurfaces, triangular patterns (from the 3-faced prism) exhibited an SPR dip at 702 nm, in close agreement with the estimated wavelength. In contrast, the square lattices (from the 4-faced prism) supported two distinct plasmonic resonances, with dips centered at 623 nm and 843 nm, consistent with the predicted response. When fabricated using the double-prism configuration, these resonances from the resulting metasurfaces red-shifted to 980 nm for the triangular lattices, while the square lattices exhibited resonances at 855 nm and 1180 nm. The origin of the additional resonance observed in the square lattices at 623 nm (single prism) and 855 nm (double prism) was further investigated using SPRi. This correlation demonstrates that the PIL technique provides direct and predictable control over the plasmonic response through geometric tuning of the interference pattern.

**Figure 11:**
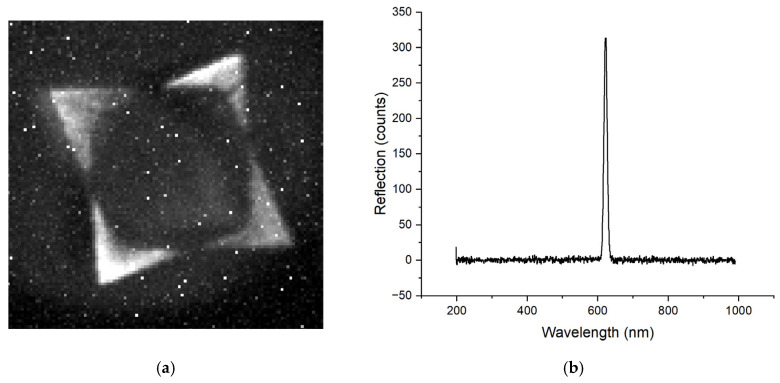
(**a**) SPRi image acquired with 623 nm light on a square metasurface fabricated using a single 4-faced pyramidal prism, showing localized plasmonic activity along the outer boundary regions. (**b**) Corresponding reflection spectrum obtained from the boundary linear grating regions, exhibiting a plasmonic resonance centered at 623 nm.

SPRi provides additional insight into the plasmonic behavior of the metasurfaces. Both the triangular (from the 3-faced prism) and the square (from the 4-faced prism) metasurfaces produced using the single-prism configuration had bright, well-defined SPRi features at their respective resonance wavelengths, indicating strong and spatially coherent plasmonic modes across the patterned regions. In the case of the square metasurface fabricated using the single 4-faced prism configuration, an additional resonance peak centered at 623 nm was observed, and when using the double 4-faced prism configuration, an additional resonance peak centered at 855 nm was observed in the reflection spectrum, as shown in Figure 9b. This resonance originates from the specific interference geometry inherent to the four-beam configuration. During the laser inscription process, two of the four interfering beams at each corner of the square lattice intercept, which produce a secondary, linear grating outside of the central square lattice. SPRi performed using the experimental set-up described in Figure 6 at 630 nm wavelength-filtered light revealed localized bright features along the outer boundary regions of the pattern, as presented in Figure 11a, confirming that the plasmonic response at this wavelength is spatially confined to these linear grating regions rather than the central square metasurface. The corresponding spectral response from the linear grating areas outside the central square lattice are presented in Figure 11b and show a clear plasmonic peak around 623 nm. This observation highlights a key strength of SPRi, where it can spatially differentiate between resonance modes originating from various metasurfaces. Such multimodal spectral signatures could potentially be exploited in future applications.

Overall, these results demonstrate that PIL enables precise and predictable control over both the structural and plasmonic properties of metasurfaces. Compared to electron-beam lithography and focused ion beam milling, PIL offers significantly reduced fabrication time and cost while enabling uniform patterning over centimeter-scale areas in a single exposure. The practical limits of PIL are primarily governed by prism geometry, laser wavelength, and photoresponsive material properties; nevertheless, the demonstrated prism- and lens-based tuning strategies provide substantial flexibility. Such tunable and spatially differentiated plasmonic responses open pathways for future applications requiring multi-resonant, spatially encoded, or broadband plasmonic functionalities.

## 6. Conclusions

This study demonstrated that pyramidal interference lithography (PIL) provides a useful and scalable method for fabricating periodic, large-area, and tunable gratings and metasurfaces. Single and double 3-faced and 4-faced prism configurations produced triangular and square metasurface structures, respectively, with the double-prism system yielding larger periodicities due to the modified interference geometry. To overcome the limitations of fabricating custom prisms, a bi-prism–lens configuration was introduced, enabling continuous and dynamic tuning of the pitch by simple axial translation of the lens.

Plasmonic characterization confirmed that the resulting metasurfaces support well-defined surface plasmon resonances, with resonance wavelengths shifting consistently with changes in periodicity. SPR imaging further highlighted the spatial distribution of plasmonic activity, including localized edge-related modes.

Overall, the results show that PIL, particularly when combined with a lens, offers a straightforward and adaptable method for producing metasurfaces with controllable periodicity and optical response, supporting its potential use in scalable photonic and plasmonic device fabrication.

## Data Availability

The data presented in this study are available on request from the corresponding author. The data are not publicly available due to privacy, and legal restrictions.

## Abbreviations

The following abbreviations are used in this manuscript:

SPR	Surface plasmon resonance
SPRi	Surface plasmon resonance imaging
PIL	Pyramidal interference lithography
